# Bone Regeneration: A Review of Current Treatment Strategies

**DOI:** 10.3390/jcm14061838

**Published:** 2025-03-08

**Authors:** Raffaella De Pace, Silvia Molinari, Elisa Mazzoni, Giuseppe Perale

**Affiliations:** 1 Department of Chemical, Pharmaceutical and Agricultural Sciences, University of Ferrara, 44121 Ferrara, Italy; 2 Industrie Biomediche Insubri SA, Via Cantonale 67, 6805 Mezzovico-Vira, Switzerland; 3 Faculty of Biomedical Sciences, University of Southern Switzerland (USI), Via G. Buffi 13, 6900 Lugano, Switzerland; 4 Ludwig Boltzmann Institute for Experimental and Clinical Traumatology, Donaueschingenstrasse 13, 1200 Vienna, Austria

**Keywords:** bone regeneration, bone grafts, orthopaedics, oncology, orthopaedica surgery, oncological surgery

## Abstract

Bone regeneration has emerged as a critical research and clinical advancement field, fueled by the growing demand for effective treatments in orthopedics and oncology. Over the past two decades, significant progress in biomaterials and surgical techniques has led to the development of novel solutions for treating bone defects, surpassing the use of traditional autologous grafts. This review aims to assess the latest approaches in bone regeneration, including autologous, allogenic, and xenogenic grafts, naturally derived biomaterials, and innovative synthetic substitutes such as bioceramics, bioactive glasses, metals, polymers, composite materials, and other specialized applications. A comprehensive literature search was conducted on PubMed, focusing on studies published between 2019 and 2024, including meta-analyses, reviews, and systematic reviews. The review evaluated a range of bone regeneration strategies, examining the clinical outcomes, materials used, surgical techniques, and the effectiveness of various approaches in treating bone defects. The search identified numerous studies, with the inclusion criteria focused on those exploring innovative bone regeneration strategies. These studies provided valuable insights into the clinical and biological outcomes of different biomaterials and graft types. Results indicated that while advancements in synthetic and naturally derived biomaterials show promising potential, challenges remain in optimizing therapeutic strategies across diverse patient populations and clinical settings. The findings emphasize the need for an integrated approach that combines scientific research, clinical practice, and technological innovation to improve bone regeneration therapies. Further research is required to establish standardized protocols and determine the optimal application of various materials and techniques to enhance patient outcomes and the quality of care.

## 1. Introduction

Bone defects caused by large tumor resections, non-healed fractures due to trauma, biochemical dysfunctions, infections, or skeletal development anomalies due to genetic disorders are among the leading causes of disability and reduced quality of life globally. The treatment of these defects to restore normal bone morphology and function represents a significant clinical challenge that has not yet been fully resolved, especially when bone healing is compromised [1]. Evidence of clinical needs related to bone reconstruction dates back to ancient Egypt and has made its way up to date. A more rigorous scientific approach has been followed since 1889, when “modern” scientists started to focus their efforts on what can be defined as the early bone tissue engineering [2]. Several surgical techniques have been developed, including the use of synthetic bone substitutes and bone grafts, to promote bone regeneration. Bone grafting is used to replace the missing bone during surgery and is a widely requested procedure [1]. Even if ten million surgical bone reconstruction interventions are performed worldwide per year [3], the current clinical gold standard for treating critical sized and non-union bone defects remains autograft bone. Being advantageous for immunocompatibility, autografts nevertheless carry a wide spectrum of risks (general anaesthesia, complex surgical manoeuvres, secondary infections, secondary fractures, pain, site morbidity, etc.) that lead to failures (more than 10%) and that are also followed by important increases in costs [4,5,6,7]. It is generally accepted that not all defects, particularly larger ones, can be addressed since few healthy sites can be harvested without loss of function [8,9]. The need for adequate bone substitutes for remodeling native bone tissue is evident and sees a wide spectrum of proposed solutions, belonging to academia, clinics, and industry [4,5,10,11,12]. Human bone is a natural composite consisting of a biopolymeric phase and a ceramic (apatite). To replicate its structure, bone implants combine a biopolymeric matrix with bioceramic reinforcement, improving the mechanical and biological properties of the implant [13]. This promotes bone healing and facilitates the transport of nutrients and oxygen [14]. Each material offers distinct advantages and disadvantages. For instance, it is challenging for a single polymer to simultaneously fulfill the requirements of optimal biocompatibility, biodegradability, porosity, and mechanical support. To address these challenges, natural polymers, synthetic polymers, cells, small molecules, and other substances are often combined [15]. Very recently, in March 2018, Winkler et al. [4] pointed out the exact situation of this arena, that calls out for improvement in currently clinically available biomaterials: “While innovative approaches have helped to unravel the cascades of bone healing, this knowledge has so far not improved the clinical outcomes of bone defect treatment. Recent findings have allowed us to gain in-depth knowledge about the physiological conditions and biological principles of bone regeneration. Now it is time to transfer the lessons learned from bone healing to the challenging scenarios in defects and employ innovative technologies to enable biomaterial-based strategies for bone defect healing”.

Since bone is the second most transplanted tissue in the world, just after blood, medical and engineering literature offer a large number of references, corroborated by industrial patents and a certain number of products on the market worldwide. Various strategies have been investigated to enhance bone regeneration, utilizing various combinations of biomaterials and biomolecules. Current trends indicate that a composite approach is the most effective for mimicking the structure of human bone [16].

Within this framework, bone substitutes can be divided into six main categories: autografts; allografts, i.e., bone segments taken from cadavers and duly sterilized; xenografts, i.e., bone segments taken from animal bones; naturally derived biomaterials; synthetic scaffolds; and composite materials.

In this narrative review, the comprehensive literature search was performed using the PubMed (MEDLINE) database up to 31 December 2024. The search strategy included a combination of the following keywords in the title, abstract, Medical Subject Heading, and keywords fields: “bone substitute”, “biomaterial”, “bone graft”, “orthopedic surgery”, “oncologic surgery”, and “bone regeneration”, excluding terms related to oral surgery, dentistry, or dental surgery. To refine the search, filters were applied to include studies published between 2019 and 2024, written in English, and focused on human subjects. The selected article types included meta-analyses, systematic reviews, reviews, and clinical trials. Studies were excluded if they lacked sufficient statistical data, were in vitro or animal studies. The studies analyzed included patients undergoing orthopaedic or oncological surgeries requiring bone regeneration. The intervention involved the use of bone grafts or biomaterials for bone regeneration. The comparison focused on different types of bone grafts, including autografts, allografts, xenograft, natural, synthetic, and composite scaffolds. The outcomes analyzed included efficacy in bone regeneration, surgical success rates, and complication rates. Results were synthesized narratively, emphasizing key findings and conclusions.

## 2. Autologous Bone Graft

Autologous grafts are freshly harvested from the patient’s own body, making them a rich source of growth factors such as BMP-2, BMP-4, fibroblast growth factor, VEGF, platelet-derived growth factor, and insulin-like growth factor 1 [17]. These grafts possess osteogenic, osteoinductive, and osteoconductive properties and include living bone precursor cells [18]. Autologous grafts offer the advantage of eliminating the risk of rejection and disease transmission since they are derived from the patient’s body [17].

The cancellous graft is the most frequently used type of autologous bone graft, it consists primarily of cancellous (spongy) bone, which is found at the ends of long bones and in the interior of other bones. Cancellous grafts, characterized by rapid revascularization, are fully resorbed within 6–12 months, so offer limited mechanical support. The induced membrane method, developed in 1986 by Masquelet, uses cancellous bone fragments to treat necrotic or infected bone tissue, improving revascularization and reducing the risk of resorption. It is effective for septic and aseptic post-traumatic non-unions and diaphyseal fractures of long bones [19,20].

Cortical grafts, a type of bone graft taken from the cortical part of the bone, which is the dense, compact outer layer, provide greater structural stability but take years to be entirely replaced by new bone. Vascularized cortical grafts, although more challenging to harvest and implant, ensure rapid healing times and high cell survival [17]. Vascularized grafts are more resistant than non-vascularized grafts during the first six weeks, as the latter undergo necrosis and resorption, while vascularized grafts heal directly through bone formation. Cortical autografts are suitable for bone defects larger than 6 cm that require structural support, but vascularized autografts are preferred for defects over 12 cm [17]. There are also bone substitutes combined with biological products, such as bone marrow aspirate (BMA) and platelet-rich plasma (PRP). Bone marrow aspirate is used for its content of mesenchymal stem cells [19]. PRP, with its growth factors, helps bone regeneration and reduces the amount of bone needed [21].

The use of recombinant human growth factors in autologous bone substitutes has shown promising results. The two engineered proteins used are recombinant bone morphogenic protein 2 (rhBMP-2) and recombinant human platelet-derived growth factor-BB (rhPDGF-BB) [22]. A recent study explored the combination of autologous iliac crest bone graft, rhBMP-2, and bone cells for treating bilateral alveolar cleft defects. The results showed more effective bone regeneration and a reduced need for additional surgeries [23]. Another study on 24 patients assessed the efficacy of autologous bone with rhBMP-2 for non-union and bone defects, reporting a fusion rate of 96% at six months and 100% at one year. Patients showed improved quality of life and reduced pain as early as three months, with no adverse effects or antibody development against BMP-2 [24].

Autografts have to be harvested from non-load-bearing skeleton region, resulting in an additional surgical site [17], and it has limitations including limited graft supply, secondary injury, donor site morbidity, chronic pain and infection [25,26].

The techniques for harvesting autologous bones significantly affect cell vitality, bone integration, and regenerative capacity. The success of autografts depends on collecting osteoprogenitor cells and vital osteocytes, but mechanical techniques or delays in implantation can compromise their efficacy [27].

Traditional approaches were developed on the principle of autografts. But ever since a couple of decades, this approach has started showing its very deep limitations, as evidenced in an always increasing spectrum of documents, including the academic literature, clinical papers, and economic market surveys. [18]

## 3. Allogenic Bone Graft

Allografts derived from both living donors’ and cadavers’ bone have been an accepted alternative to autograft. They are prepared by removing the soft tissue with ethanol and then sterilized [17]. Allografts can be implanted in three forms: fresh, fresh-frozen, and freeze-dried [19]. They can be divided into three different categories: cancellous allograft, cortical allograft, and demineralized bone matrix.

The cancellous allograft, made of freeze-dried cuboid bone fragments, includes benefits like low residual moisture (3–5%) and a shelf life of 4–5 years. Like its autologous cancellous counterpart, cancellous allografts also lack mechanical strength. However, while the cancellous autograft has several osteogenic properties, the cancellous allograft only provides osteoconduction. Another downside is that the host’s inflammatory response surrounds the graft with fibrous tissue, making its integration more difficult [17]. Structural allografts are commonly used in the treatment of acute fractures and revision traumatic reconstruction surgery. Berkes et al. [28] published a review of 77 patients who underwent Open Reduction and Internal Fixation (ORIF) of Schatzker II tibial plateau fractures with structural bone allografts. All patients united with good functional outcomes and a 0% subsidence rate, meaning there was no sinking or movement of the bone graft in the fracture area. A study by Fujii et al., which included a total of 64 participants and a 10 year follow-up, suggests that transposition osteotomy of the acetabulum (TOA) with structural bone allografting is a valid surgical option for correcting severely dysplastic acetabulum in adolescents and young adults, with favorable mid-term results [29].

The osteochondral grafts are fresh or frozen, used more frequently in post-traumatic reconstructions rather than in the acute traumatic setting. The use of osteochondral grafts has been reported in acute trauma situations, such as in a severe fracture-dislocation of the elbow, where the radial head was transplanted. The clinical results were satisfactory, with the patient returning to heavy-duty activities four years after the surgery and showing only mild radiographic degeneration [30].

Allogenic bone grafts have a higher failure rate compared to autologous grafts because they are immunogenic and may be rejected due to the activation of major histocompatibility complex antigens. They also exhibit reduced osteoinductive properties, toxicity associated with sterilization methods, risk of immunoreactions and transmission of infections [17]. Another concern with the use of allografts is that their demand is far exceeding supply, despite an increase in the number of cadaver donors [31]. Moreover, these concerns have led to an increase in regulatory restrictions on the use of these materials.

The systematic review by Moraschini et al. aimed to evaluate the immune response to allogenic bone graft procedures in humans. The studies report that an average of 48% of patients remain immunologically sensitized after receiving allogenic bone grafts. Future studies should better address the local and systemic consequences of this sensitization [32]. While modern techniques such as sterilization and freezing significantly reduce the risk of transmitting infectious agents in allogeneic bone grafts, these processes can also compromise graft performance such as osteoinductive potential, bone conduction, and mechanical strength [33], resulting in a lower rate of incorporation within the bone [34].

With ongoing advancements in preparation techniques, lyophilized allogenic bone has become a widely used form of allogenic graft. Recent studies have demonstrated that decalcified lyophilized allogenic bone retains growth factors that enhance bone induction, facilitating the aggregation of mesenchymal stem cells (MSCs) and promoting osteoblast differentiation [35]. The study by Clark et al. evaluated the use of decalcified freeze-dried allogeneic bone for dorsal nasal augmentation. The dorsal augmentation was considered successful in 85 percent of the 62 patients evaluated [35]. A randomized clinical trial assessed the use of freeze-dried bone allograft blocks for lateral maxillary ridge augmentation. The results indicate that these grafts provide a clinically acceptable alternative for bone reconstruction [36]. It has been the only biomaterial approved by the Food and Drug Administration (FDA) in North America for clinical bone repair [37].

Demineralized bone matrix (DBM) is a type of allograft in which an acidic solution is used to extract the mineral content while preserving most of the bone’s protein structure. It retains small traces of calcium-based solids, inorganic phosphates, and minimal cell debris. DBM functions as both an osteoconductive and osteoinductive material and is officially recognized as a medical device for applications in bone defect repair and spinal fusion procedures [38]. A narrative review by Ren et al. presents numerous studies that support the therapeutic application of DBM in periodontal intraosseous defects, sinus lift procedures, crest preservation, crest augmentation, alveolar fissure repair, orthognathic surgery, and other regional maxillofacial bone defects [39]. Manawar et al. highlights how DBM has been successfully used in various spinal surgeries, in the treatment of bone cysts, nonunion of long bones, and in cases of small bone defects in the foot. DBM has also been studied in relation to tibial lengthening, showing a significant improvement in the bone consolidation process [40].

DBM products have been increasingly used in clinical applications, and some therapeutic effects have been achieved. However, there are still several problems that need to be studied and discussed some: (i) osteoinductive properties are lost during processing and sterilization [41]; (ii) the powder or particles of DBM can be loose in structure and may not stay firmly in the filling site, and could be easily dispersed by blood flow during surgery [42]; (iii) there is currently no DBM product that can combine the various conditions of ideal bone graft materials; and (iv) different materials, different methods, different reagents, and even the same conditions of batch processing of DBM products have differences in osteogenic activity [38].

## 4. Xenogenic Bone Graft

Xenografts are derived from animal species, are osteoconductive, relatively inexpensive, do not lengthen the healing time, and the need for a second surgical site for bone harvesting is eliminated [43].

The first bone xenotransplantation dates to 1668, when a bone fragment from a canine skull was transplanted into a human patient in Russia. Later, in 1957, Maatz and Bauermeister documented the transplantation of bovine bone [44]. To prevent rejection mediated by the alpha-Gal epitope (a carbohydrate structure found on the surface of many non-human mammalian cells), which is recognized as foreign by the human immune system, xenografts are decellularized to reduce epitope levels and create a suitable biological scaffold [44]. This process does not compromise the graft’s strength, which is essential for osteoconduction, nor eliminates the osteoinductive growth factors embedded in the extracellular matrix [45]. Decellularized xenograft bone scaffolds provide a unique natural microstructure that not only has stiffness comparable to human bone tissue but also features similar components, porosity, and pore size. Decellularized xenograft cancellous bone appears to be the best alternative to autografts, as this scaffold supports capillary growth, osteoblast migration and proliferation, and osteogenesis. Its availability, cost-effectiveness, and ability to be produced in a wide variety of geometric shapes make it suitable for mass production [46]. 

Over time, several studies have examined the use of bovine bone substitutes in orthopaedics and various commercial xenograft products are reported in the orthopaedic literature (Table 5). CANCELLO-PURE^®^ is a xenogeneic bone graft derived from bovine, processed through decellularization to reduce the risk of immune rejection. It has been used in foot and ankle reconstruction procedures, such as Cotton and Evans osteotomy [44]. However, some studies have reported high rates of graft non-union and failure to integrate, leading to its discontinuation in certain institutions. Kiel Surgibone^®^ is a bovine bone graft treated with specific methods to remove antigenic components while preserving the bone’s mineral structure. It has been used in spinal fusion surgeries, such as anterior cervical discectomy and fusion (ACDF), showing fusion rates comparable to autografts in various clinical studies [44]. BIO-GEN^®^ is a heterologous bone substitute obtained from equine bone, processed through deproteinization and sterilization to remove antigenic components. It is used in oral and maxillofacial surgery for guided bone regeneration, supporting new bone formation thanks to its porous structure and biocompatibility [47].

The availability of xenogeneic bones is unlimited despite the possibility of zoonosis transmission if they can be handled for the host [48].

It was mentioned that other factors like the implanted site location (where the bone graft is placed in the recipient’s body) and epidemiological parameters (factors related to the recipient’s health, immune status, or potential exposure to certain diseases) influenced the osteogenic ability of xenogeneic materials [37].

## 5. Naturally Derived Biomaterials

Naturally derived biomaterials such as collagen (Col), gelatine (Gel), alginate (Alg), fibrin, silk fibrin (SF), hyaluronic acid (HA), polyhydroxyalkanoate (PHAs), and chitosan (CS) are produced by living organisms and degraded into carbon dioxide and water [26]. Natural polymers are extensively used in tissue engineering due to their natural biocompatibility and biodegradability; however, they often lack the required mechanical properties [2,8,9,49]. For details on advantages and disadvantages, refer to Table 1.

### 5.1. Col, Gel, and Alg-Based Materials

Col, a key component of natural bone, is widely used in bone regeneration in forms like membranes, sponges, and hydrogels [50]. Col membranes are extensively applied in guided bone regeneration (GBR) for improved soft tissue healing and host tissue integration, despite limitations in mechanical strength and resorption rates [51]. Col sponges, valued for their processability and bioactivity, are used in bone tissue engineering (BTE) for bioactive substance delivery, with promising results in BMP-2-enhanced spinal fusion [52] and FGF-2/BMP-2 gene delivery systems [53]. Col hydrogels, known for biocompatibility, face limitations in stiffness and degradation [54], mitigated by composite hydrogels incorporating functionalized polymers, nanoparticles, and ions to enhance mechanical properties and stem cell adhesion [55]. These materials are used in innovative applications like injectable scaffolds, drug delivery systems, and 3D-printed or electrospun scaffolds for bone repair [56].

Due to its biocompatible and biodegradable properties, Gel is widely used in bone regeneration but is most often combined with other materials to enhance scaffold performance [57]. Liao et al. demonstrated that the addition of nano-hydroxyapatite to macroporous Gel scaffolds via cryogelation increases toughness and porosity [58]. Similarly, Gel coating on PCL/graphene oxide and scaffolds improves hydrophilicity, degradability, and hydroxyapatite deposition, promoting cell adhesion [59].

The use of Alg in BTE has been widely documented, and Alg polysaccharides have been approved by the U.S. FDA as safe for human use [60]. Alg-based hydrogels, obtained through Ca²⁺ crosslinking, have been extensively studied in the biomedical field. They are used as scaffolds for delivering bioactive molecules or cells, particularly in applications related to cartilage and bone tissue regeneration. Compared to traditional surgical techniques, these materials can be injected in a minimally invasive manner into the affected area of the patient, enabling the treatment or repair of damaged bones. Moreover, they are easily chemically modifiable and allow for controlled release of tissue-inducing factors such as BMP and TGF-β [61]. Col, Gel, and pure Alg have certain disadvantages that limit their applications in biomedicine and BTE. The main issue lies in the unpredictability of the degradation rate of natural materials. Rapid degradation, as in Gel and Col-based materials, and slow degradation, as in Alg-based materials, can interfere with bone healing. Rapid degradation can compromise the structural integrity of the material before new bone has formed, preventing proper fracture ossification. On the other hand, too slow degradation can delay the replacement of the material with new bone tissue, causing complications such as chronic inflammation or failure of the material to integrate with the surrounding bone. Further studies are needed to overcome existing limitations and optimize their clinical application.

### 5.2. SF, HA, CS, and PHA-Based Materials

Silk scaffolds are developed in various forms and structures to adapt to different types of tissue, such as skin, bone, cartilage, and blood vessels. It is worth noting that, despite the excellent mechanical properties of natural SF fibers, most silk-based materials developed from silk solutions are fragile [62]. To date, there is no clinical application of silk scaffolds for musculoskeletal systems as they do not meet the requirements of musculoskeletal system regeneration, such as the mechanical requirements of human daily life and exercise of tendons. At present, the research on silk scaffolds is still limited to animal experiments, and even large animals have relatively few studies [63].

HA is a natural polysaccharide. It is used in bone tissue engineering, usually combined with various polymers to improve its poor mechanical properties and low tensile and compressive strength [64]. A material based on HA, Gel, and BCP granules has shown mechanical strength similar to that of a cancellous bone substitute, while also demonstrating excellent cell growth, proliferation, and bone tissue regeneration [65]. Another material based on HA, silk fibroin, hydroxyapatite, and the hexapeptide glycine–alanine–glycine–alanine–glycine–X (where X can be tyrosine, serine, or valine) has shown a porous structure with optimal mechanical properties and good biocompatibility. This combination of materials promotes osteogenesis and exhibits antibacterial properties against S. aureus and E. coli [66]. However, there are still some challenges to address before HA-based biomaterials can be translated into clinical applications, including optimization of mechanical properties and bioactivity [67]. 

CS is a natural polysaccharide derived from chitin, widely studied for its potential applications in tissue engineering. Various CS-based materials have been designed and reported in the literature. For example, CS nanofibers are a promising material due to their high surface area and porosity, which enhance cell adhesion, proliferation, differentiation, and mineralization [68]. The addition of carbon-based materials increases mechanical strength of CS-based biomaterials [69]. For example, HAp nanoparticles in CS scaffolds strengthen the structure and stimulate mineralization [70]. CS-based scaffolds are used in drug delivery systems for prolonged and controlled release. They can carry growth factors, polyphenols, antibiotics, statins, bisphosphonates, and anti-inflammatory agents, enhancing their bioavailability. Modifications of CS functional groups optimize drug loading and enable targeted and site-specific delivery [71].

Polyhydroxyalkanoate (PHA) are materials synthesized by bacteria. They have the advantage of bioactivity but the disadvantages of high batch to batch variation [26]. PHA and its derivatives, such as PHB (polyhydroxybutyrate), PHBV (polyhydroxybutyrate-co-valerate), P4HB (poly-4-hydroxybutyrate), PHBHHx (polyhydroxybutyrate-co-hexanoate), and PHO (polyhydroxyoctanoate), are promising materials for bone tissue regeneration. A distinguishing feature of these materials is that during their degradation, the local pH of PHA scaffolds remains stable, ensuring good tolerance by the immune system [72]. Studies have reported positive results on the use of PHB and PHBV in both in vitro and in vivo approaches to bone regeneration. PHBHHx has been used in various forms, such as micro-channeled membranes, aligned nanofibers, or composite materials loaded with carbon nanotubes. These configurations have demonstrated the ability to support the osteogenesis of human mesenchymal stem cells [72]. However, further research is required to explore their clinical feasibility, considering aspects such as the degradation behavior of the scaffolds [73].

### 5.3. Natural-Based Hydrogels, Nanofibers, and Composite Materials

Col, CS, and Gel have mechanical limitations that restrict their direct application. Theoretically, these limitations could be overcome by combining these biomaterials with other materials or reinforcing agents, or by synthesizing them in different forms, such as hydrogels, nanofibers, or composite structures [74]. Gharati et al. [75] investigated Col-based hydrogel nanocomposites with 2% strontium (Col/BGSr2%) in both in vitro and in vivo studies, demonstrating high osteogenesis. Nabavi et al. [76] explored the use of type I Col hydrogels enriched with tacrolimus, a macrolide antibiotic, which enhanced osteogenic differentiation by activating BMP receptors. Both in vitro and in vivo analyses revealed that hydrogels provided a supportive environment for cell proliferation and significantly facilitated bone healing processes. Han et al. [77] developed a gelatin-based hydrogel incorporating silver nanoparticles (AgNP) to promote bone regeneration and aid in fracture repair. Their findings revealed that the AgNP/Gel hydrogels were non-toxic to osteoblasts and enhanced cell viability. Several studies have used CS in combination with other substances. Kaur et al. [78] developed injectable chitosan-collagen hydrogels (CS/Col) combined with carboxyl-functionalized single-walled carbon nanotubes (COOH-SWCNT) and sodium β-glycerophosphate as a thermoresponsive cross-linker. This enhanced mechanical properties, cell proliferation, and hydroxyapatite formation within 24 h. Furthermore, CS and Col hydrogels, such as those with carbon nanotubes, have shown good biocompatibility and thermoresponsive properties for bone applications [78]. Despite promising results in vitro and in vivo, it is crucial to further evaluate and validate these approaches in clinical environments to ensure their safety and effectiveness for bone tissue engineering applications [74].

## 6. Synthetic Bone Grafts

Synthetic bone grafts are available in various forms, including moldable, pellets, injectable, and 3D printed [17]. A number of synthetic materials are clinically available to stabilize and bridge large bone defects including metals (e.g., stainless steel), bioglass, bioceramics (e.g., hydroxyapatite, tricalcium phosphate, and bioactive glasses) and polymer organic (e.g., polylactic acid, polylactic-co-glycolic acid, and polycaprolactone).

The moldable and pellet forms of grafts are advantageous in trauma or when surgery is not preplanned to 3D print a graft [17]. Schmidlin et al. compared two synthetic, moldable calcium phosphate ceramics (CaP) materials in a cranial defect model. The results showed that materials were biocompatible and osteoinductive: after four weeks, the treated defects exhibited a high rate of bone regeneration, and after 16 weeks, the materials were partially resorbed. Moldable synthetic CaPs are safe and suitable bone graft substitutes [79]. Researchers claim that calcium sulfate pellets provide an effective void filler, allow vascular growth, and are rapidly and completely resorbed, enabling physiological bone healing [80]. Injectable forms represent an excellent option as they allow for minimally invasive surgery. In the last four decades, calcium phosphate ceramics (CaP) have been widely used as injectable materials for orthopaedic surgery due to their excellent properties in terms of biocompatibility and osteoconductivity [81]. A study analyzed the injectability of a CaP scaffold for the first time. Processing parameters were optimized to ensure high injectability, macroporosity, and mechanical strength similar to cancellous bone and sintered porous hydroxyapatite implants [82]. 

Three-dimensional bioprinting represents a significant innovation in the production of customized bone grafts, offering tailored solutions to meet the specific needs of patients. This technology allows the creation of structures that mimic the composition and function of natural bone, enhancing tissue integration and regeneration [83]. In the article by Chung JJ et al., 2020, different types of 3D printing techniques used for bone tissue regeneration have been listed and compared in order to underline advantages and limitations [84]. A practical example of this technology is the use of CMFlex, a 3D-printed synthetic bone graft developed by Dimension Inx. It has been successfully implanted in patients, demonstrating the effectiveness of 3D-printed bone grafts in medicine [85]. Furthermore, the Rizzoli Orthopaedic Institute in Bologna uses “custom-made” prosthetics made from trabecular titanium via 3D printing. These custom prosthetics enable precise anatomical reconstruction, improving the functionality and fit of the implant for the patient [86]. These developments highlight the potential of 3D bioprinting in revolutionizing the production of bone grafts, offering customized solutions and improving clinical outcomes for patients. Despite the extensive research on 3D printing for biomedical applications, there is still significant potential for further enhancement [87,88].

### 6.1. Bioceramics

Bioceramics have been extensively studied for treating bone defects due to their favorable biocompatibility, biodegradability, osteoconductivity, and osteoinductivity. However, bioceramic powders cannot be directly used for bone repair due to their rapid degradation and loss of volume. To overcome this limitation, various three-dimensional porous scaffolds have been developed, which stimulate the growth of internal bone tissue and enable the effective use of bioceramics in bone defect treatments [89].

Bioceramics include CaP, i.e., tricalcium phosphate (TCP), hydroxyapatite (HAp), biphasic calcium phosphate (BCP), and calcium sulfate bone cement. Advantages and disadvantages of these biomaterials are described in Table 2.

CaP are synthetic materials with high crystallinity, produced through sintering at temperatures above 1000 °C. They have an inorganic composition similar to natural bone and are useful as scaffolds for bone repair [17]. Osteoinductivity of these biomaterials can be increased through surface engineering. Modifications such as introducing pores, appropriate morphology, and/or increased roughness can enhance the concentration of specific proteins or cytokines, the recruitment of immune and osteoprogenitor cells, and improve cell adhesion, proliferation, and differentiation into osteoblasts, thereby stimulating bone formation [90].

HAp bioceramics are commonly used for bone tissue replacement, especially in porous or pellet forms. The pores in HAp enable both chemical and biological bonding with the bone, promoting stable fixation of the implant and preventing loosening [89]. Commercially available porous HAp implants have relatively low mechanical resistances, but these significantly improve once the pores are filled with natural bone tissue. When the pores are filled 50–60% with cortical bone, the bending strength can increase to 40–60 Mpa [91].

HAp has some shortcomings including low degradability and fragility [90,92,93,94,95,96]. The slow resorption of bioceramics can delay the bone remodeling process, making the bone more mechanically vulnerable [17]. To mitigate this issue, nanocrystalline HAp has been developed, offering a higher surface-to-volume ratio that promotes faster resorption and potentially accelerates bone healing. Additionally, the production of nanocrystalline HAp is simpler because it requires a lower sintering temperature [97]. Regarding spine surgery, HAp is frequently used as a laminar spacer in cervical laminoplasty. Bone in growth has been seen at the interface between the opened lamina and laminar spacers used for double-door laminoplasty due to their good osteoconductivity [98].

Numerous synthetic HAp-based materials are commercially available: Apaceram^®^ [99,100], Straumann^®^ BoneCeramic™ [101], Cerament^®^ Bone Void Filler [102,103], and Nanostim^®^ is a form of nanocrystalline HAp [104]. Their characteristics and main clinical applications are described in Table 5.

TCP is particularly effective in treating bone defects caused by trauma or tumors [105]. However, due to its low tensile strength and fragility, it is generally avoided in load-bearing defects and areas subjected to high mechanical loads, where materials with greater structural strength may be required [106]. There are two types of tricalcium phosphate, namely: α-tricalcium phosphate (α-TCP) and β-tricalcium phosphate (β-TCP). The α-TCP has a monoclinic crystal structure, while the β-TCP phosphate has a rhombohedral crystal structure [107]. Regarding β-TCP, it is more widely used than α-TCP, because it is more stable and has a higher biodegradation rate [108]. On the other hand, the β-TCP scaffolds degrade too quickly. This means the material breaks down and dissolves before the newly formed bone can adequately replace it [109]. Synthetic β-TCP-based materials are used for repairing fractures or bone defects, filling cavities caused by bone loss, and promoting the formation of new tissue. They are also employed in treating lesions due to congenital malformations or degenerative diseases [110]. Among the most common materials KeraOs^®^ is used in orthopaedics for bone regeneration, particularly in cases of bone defects and fractures [104]. Vitoss^®^, with its porous structure, is used in orthopaedics to stimulate bone growth in complex fractures or significant bone defects [111]. SynthoGraft™ is utilized in orthopaedic surgical procedures requiring safe and gradual regeneration of compromised bone tissue [112,113] (Table 5).

BCP is created by combining HAp with TCP to leverage the benefits of both materials. By modifying the ratio of HAp to TCP, it is possible to achieve varying resorption rates and mechanical properties, allowing for customization to meet specific clinical needs. This combination of resorption times and mechanical stability ensures that the material remains stable while promoting bone growth. Thanks to these properties, a BCP bone graft is suitable for use in large bone defects and weight-bearing regions [17]. Numerous commercial products are available on the market: Maxresorb^®^ [114], MBCP+ (Micro-Macroporous Biphasic Calcium Phosphate) [115], Ceraform^®^ [116]. Their characteristics and main clinical applications are described in Table 5.

Calcium sulfate dissolves quickly in 4–12 weeks, making it the fastest dissolving material, but this rapid degradation can hinder bone regeneration. It has limited osteoconductive capacity due to low porosity and is less commonly used today, as calcium phosphate-based materials are preferred. Additionally, it loses mechanical properties during degradation, making it unsuitable for load-bearing areas. Its advantages are low cost and ease of preparation [17]. Bondbone^®^ is a commercial synthetic biphasic calcium sulfate material used in orthopaedic applications [117]. EthOss^®^, composed of β-TCP and calcium sulfate, is frequently used to reconstruct complex bone defects [118] (Table 5).

### 6.2. Bioactive Glass

Bioactive glass (BG) or bioglass, a silica-based material, is widely used as synthetic bone grafts. The material stimulates the patient’s body regeneration capabilities and it has antibacterial properties due to its alkaline composition [17], but it has limited degradation [109], and low mechanical properties that restrict its use in load-bearing regions [119,120,121,122,123]. Compared to other synthetic bone grafts, BG is different as it can form chemical bonds to bone and soft tissue [17].

The FDA approved the first bioactive glasses in 1985, and since then, numerous devices have been approved for various medical applications [124]. At least 25 BG medical devices have been approved for clinical use, with applications in bone implants to wound and cancer treatments. Technology has evolved from early glasses like 45S5 Bioglass^®^, with a reduction in particle size and greater variety in morphologies (from monoliths to granules and cements), improving flexibility for professionals. The main compositions are based on silicate, but borates and phosphates have also been explored for tissue regeneration [124].

Some examples of BG used for bone tissue regeneration, currently on the market, used for bone tissue regeneration are: NovaBone^®^ [125], Medpor^®^-Plus [126], BoneAlive^®^ uses S53P4, a silicate-based composition, with a slower dissolution rate compared to 45S5 [127], Glassbone^®^ [128], Cortoss^®^ [129], StronBone™, a strontium-doped bioactive glass [130], Glace^TM^, a fiber-glass [131], OssiMend^®^ Bioactive, combining 45S5 bioactive glass, type 1 Col, and carbonated apatite [132,133]. Signafuse^®^, a composite of bifasic minerals and BG [134]. Their characteristics and main clinical applications are described in Table 5.

Research on the use of BG in spinal fusion has been initiated to reduce pseudoarthrosis rates while protecting against post-operative infections. Combining BG with local autograft has shown improved clinical outcomes without negative effects on infections or immune responses [135]. In a study involving 396 patients, 84% achieved successful spinal fusion with extended BG autografts. Additionally, including BG in spinal fusion grafts reduced infection risk and led to pain reduction. A new clinical study, started in 2021 at Turku University Hospital, is investigating the use of BonAlive^®^ putty for spinal fusion, with results expected in 2023 [128].

Fiber-reinforced composites containing bioactive glasses have been widely used in maxillofacial surgery and cranial implants, showing success in bone defect repairs and other applications [136].

In the treatment of osteomyelitis, bioactive glasses like S53P4 have shown positive effects. In a 2018 study, 50 patients with chronic osteomyelitis were treated with a BG formulation after surgical debridement. A total of 70.3% of patients healed after 6 months, and 83.3% healed after 12 months without the need for antibiotics, thanks to the natural antibacterial properties of BG [137].

The main challenge of bioactive glasses for bone grafting is controlling the material’s dissolution rate. Too rapid a dissolution could interfere with bone healing, while too slow a dissolution could hinder the integration of the material with the surrounding bone. Studies have highlighted the need to optimize dissolution to promote bone regeneration without negative side effects [138]. Another significant challenge is ensuring biological compatibility. Research focuses on analyzing biocompatibility and minimizing potential toxic effects to ensure that the use of bioactive glasses does not pose health risks to the patient [139].

### 6.3. Metallic Materials

In the 1950s, Charnley developed a stainless-steel joint that achieved great success in clinical practice [140]. Stainless steel, while strong, has a much higher elastic modulus than human cortical bone. This means it absorbs too much mechanical stress, reducing the load on the bone (stress shielding), which in turn decreases the mechanical stimulation needed to maintain bone health, promoting osteoporosis [141]. To overcome these issues, titanium (Ti) and cobalt (Co)-chromium (Cr) alloys have become the preferred choice due to their good biocompatibility, mechanical properties, improved corrosion resistance, and elasticity closer to that of bone, reducing stress shielding [142]. Furthermore, these implants often require a second surgery for removal after bone healing, increasing surgical risk and patient discomfort [141,143]. To avoid secondary procedures, biodegradable metallic materials have been developed, which gradually degrade until they disappear after healing. Among these, magnesium (Mg) alloys are particularly valued in the biomedical field for good mechanical properties similar to natural bone, excellent biocompatibility, biodegradation, and osteogenesis. Mg and its alloy implants have a broad application prospect in fracture fixation [37]. Iron (Fe) alloys are considered biodegradable materials because, in humid environments such as the human body, they gradually oxidize, decomposing without leaving harmful residues in the body [144]. Also, Zinc (Zn) alloys provide stable mechanical support and gradually dissolve without leaving residues. However, challenges remain, such as the mismatch between the metal degradation rate and bone healing speed, as well as the loss of mechanical strength due to rapid degradation [145]. Bismuth (Bi)-based alloys, with a low melting point around 60 °C, can be injected into bone cavities and solidify in situ, making them better suited for clinical needs [146]. Finally, magnetostrictive iron-gallium (Fe-Ga) alloys are being studied for their ability to deform under magnetic fields, opening new possibilities for intelligent implants [147]. Beyond traditional and biodegradable metals, new materials with unique properties have emerged. Porous tantalum (Ta) is highly corrosion-resistant and has an elasticity similar to that of bone, making it ideal for orthopaedic implants [148]. Ta is an inert metal with anticorrosive properties [109]. Ta scaffolds are designed with a porous structure to better match natural bone. This helps reduce stiffness and increase elasticity, making them more similar to human bone. However, the production of these scaffolds is complex, and the process of bone formation around them occurs slowly, which limits their clinical use [26]. The characteristics and uses of these metallic materials are summarized in Table 3.

Three-dimensional-printed metallic implants are revolutionizing spinal surgery. The main applications include internal fixations (to stabilize the vertebrae), spinal cages (to support the spine), vertebral replacements (to replace damaged bones), and artificial discs (to replace damaged intervertebral discs) [149]. Clinical studies have shown their effectiveness. Thayaparan et al. [150] designed a 3D-printed Ti posterior fixation implant to treat three women with atlantoaxial osteoarthritis, achieving good results without damage to nerves or blood vessels. Additionally, they developed a custom-made Ti implant to stabilize the connection between the skull and cervical spine, with good patient recovery after six months [151]. Mróz et al. [152] studied new 3D-printed intervertebral disc implants, made from a cobalt-chromium-molybdenum alloy using laser 3D printing technology. These implants showed the ability to restore the natural range of motion of the spine and the height of the intervertebral disc. Three-dimensional-printed joint implants have shown good outcomes also in shoulder, hip, knee, and ankle surgery [153]. In total hip arthroplasty (THA), the use of customized metal scaffolds has proven to be highly beneficial. Geng et al. [154] analyzed 92 patients and found that the 3D-printed porous Ti acetabulum provides excellent stability due to its interconnected structure. For patients with severe bone loss undergoing revision total knee arthroplasty (TKA), Ao et al. [155] demonstrated that 3D-printed porous Ta cones are effective in reconstructing bone defects and providing good anatomical support. Follow-up X-rays showed that the implants were stable and that the bone defects had been properly reconstructed. Regarding total ankle arthroplasty (TAA), outcomes are generally less satisfactory compared to those of the hip and knee. However, Faldini et al. [156] tested the use of 3D-printed Co-Cr metal components, demonstrating good implant positioning in postoperative X-rays. Zhang et al. [157] found that 3D-printed porous Ta offers excellent biocompatibility and promotes bone growth in patients with ankle osteoarthritis. In oncologic surgery, 3D-printed metallic implants significantly improve the treatment of bone tumors, helping to restore both function and appearance of the bone [153,158]. Chen et al. [159] described the case of a 29-year-old woman with a giant chondrosarcoma, treated with a customized 3D-printed Ti implant. The surgery was successful, resulting in significant pain relief and good functional recovery after the procedure. For the treatment of bone tumors in the knee, Luo et al. [160] designed a 3D-printed titanium alloy (Ti–6Al–4V) implant for partial knee replacement following tumor resection. In all four cases examined, the implant fit the bone defect precisely. The use of 3D-printed metal prostheses can improve the treatment of complex post-traumatic skeletal defects and deformities [153,161]. For open fractures of the distal humerus with severe bone loss, Luenam et al. [162] used 3D-printed metal powders to create customized implants. After 24 months, X-rays showed good joint spacing, indicating a successful outcome. For patients with early-stage osteonecrosis of the femoral head, Zhang et al. [163] developed a 3D-printed trabecular Ti bone implant to replace necrotic bone. The results showed that this implant could delay disease progression. Zhao et al. [164] successfully treated a nonunion tibial fracture using a porous Ta metal plate, demonstrating excellent mechanical and biological properties. Kadakia et al. [165] described the use of a 3D-printed cage to provide structural support in patients with collapsed talus and a large bone defect. Traditional metallic fixation devices often cause osteopenia and weaken surrounding healthy tissue, because the elastic modulus of metals is greatly higher than the surrounding tissue [166]. Thus, metals are suitable to be applied in load-bearing bone defects but pose a high risk of stress shielding [25]. Metal alloys need further improvements for more effective clinical use.

### 6.4. Polymer Organic Synthetic Materials

Synthetic organic polymers offer several advantages, such as controlled molecular weight, ensuring minimal variation between batches and uniformity and reproducibility in clinical applications [107]. Additionally, their composition and microstructure can be modified, enabling customization of their physicochemical properties. The presence of aliphatic ester bonds makes these biopolymers susceptible to hydrolysis, facilitating their natural degradation [107]. However, the significant limitation is their bioinertness and absence of bioactivity, which reduce their effectiveness in bone implant applications, where it is crucial to promote integration with the surrounding tissue. To overcome this obstacle, supplementary bioactive materials are often incorporated, improving the interaction between the polymer and bone tissue [107]. Most commonly used synthetic polymers are polymethyl methacrylate (PMMA), polylactic acid (PLA), polyglycolic acid (PGA), polylactic-co-glycolide (PLGA), and polycaprolactone (PCL) [167] (Table 4).

PMMA is a thermoplastic polymer known for its good biocompatibility and mechanical properties. It has been extensively used in spinal vertebroplasty and as a bone filler in patients with primary tumors or metastases. However, its application raises several health concerns [168,169,170]. PMMA tends to fragment easily, which can trigger foreign body reactions, resulting in prosthesis loosening and osteolysis [171]. As a result, new applications for PMMA have been developed, including its use in composite materials and 3D printing of customized bioimplants [172,173,174,175].

PMMA–CaP-based scaffolds and PMMA-BODBB represent two promising approaches for bone regeneration, with enhanced biocompatibility, osteogenesis, and mechanical strength of PMMA. PMMA–CaP, enriched with 60S bioactive glass, are designed with a highly porous structure and good structural interconnectivity. The integration of bioactive glass improves bone formation due to the material’s mechanical stability and osteogenic properties [176]. Il PMMA can be synthesized through a radical polymerization process, often catalyzed by benzoyl peroxide (BPO). However, PMMA catalyzed by BPO exhibits disadvantages including an exothermic polymerization reaction and a lack of bioactivity. PMMA-butoxydibutylborane (BODBB) uses BODBB as a catalyst. This reduces the risks of exothermic reactions and enhances the material’s bioactivity, cell adhesion, proliferation, and osteogenesis, due to the reduced release of free radicals and toxic monomers, along with the controlled release of bioactive boron. In a rat cranial bone defect model, PMMA-BODBB showed the highest level of osteointegration [177]. The addition of tantalum carbide (TaC) to PMMA improves its mechanical, radiopaque, and osteogenic properties without compromising biocompatibility. The TaC-PMMA composite has a compressive strength greater than 100 MPa and a radiopacity similar to that of commercial bone scaffold, offering a potential enhancement for orthopaedic applications [178].

However, studies on this material have been limited to animal models [179]. However, clinical studies that confirm the results obtained in in vivo models are necessary, to ensure their effectiveness and safety in clinical applications [176].

PGA is a synthetic polymer used in internal bone fixation due to its biodegradability, good mechanical properties and biocompatibility [180]. Research has shown that membranes made from PGA can support bone regeneration in guided bone regeneration (GBR) procedures [181,182]. Furthermore, PGA exhibits excellent thermal stability and a high melting point, making it well-suited for sterilization processes [183]. The degradation mechanism of PGA in the body occurs through the hydrolysis of glycolic acid (GA). Increased levels of GA can lead to elevated local acidity, which causes inflammation and damage to the surrounding tissues. To improve mechanical characteristics and control degradation times, PGA is frequently combined with other polymers, such as PLA, to form copolymers like PLGA [107].

PLA is a biodegradable polymer. PLA membranes used in GBR, prevent soft tissue cells from invading the damaged bone area, promoting the growth of new bone tissue [181]. These polymers are currently utilized in orthopaedic applications for implantable devices with low mechanical load, such as screws and plates for bone fracture fixation. However, their widespread use is still restricted due to their limited mechanical strength [184]. PLA has some limitations, such as hydrophobicity, low impact toughness and slow degradation, which can cause long-term inflammation, and the release of acidic byproducts, increasing the risk of adverse reactions [26,185]. To address these issues, PLA copolymers and composite materials are developed, enhancing their compatibility and degradation rate [186].

PLGA is a biodegradable and biocompatible copolymer composed of PLA and PGA, widely used in drug delivery and tissue engineering. The degradation rate can be adjusted by modifying the ratio between the two acids in the copolymer [181]. PLGA scaffolds provide physical support and a surface for cell adhesion and proliferation, promoting new bone tissue formation [187]. PLGA limitations, including poor mechanical strength, the release of acidic byproducts, hydrophobicity, and suboptimal bioactivity, pose significant challenges to its applications [188]. To overcome these drawbacks, several studies have focused on combining PLGA with inorganic materials to improve its overall properties [188]. Sheikh et al. [189] and Selvaraju et al. [190] developed hybrid PLGA scaffolds combining silk fibroin-Hap (SF/Hap) and collagen-Hap (Col/Hap), improving hydrophilicity, mechanical stability, and cellular infiltration, with successful in vivo ossification. Pelaseyed et al. [191] designed PLGA-TiO₂ scaffolds, which exhibited enhanced porosity, bioactivity, and cell adhesion, while also slowing down polymer degradation.

Among the currently available PLA-based products, LactoSorb^®^ is widely used in osteosynthesis for maxillofacial surgery [192]. Neoveil^®^ is a 100% PGA membrane, used for GBR, in oral surgery and implantology [193]. Among PLGA-based materials, Ethisorb^®^ is a fixation device for maxillofacial and orthopaedic surgery [194]. Another significant product is RapidSorb^®^, employed in cranio-maxillofacial fixation, with a particular application in post-surgical stabilization for pediatric procedures and reconstructive surgery [195]. Their characteristics and main clinical applications are described in Table 5.

Polycaprolactone (PCL) is a polymer with high mechanical strength and is used as a scaffold for bone and periodontal tissue engineering [196]. PCL can be transformed into porous scaffolds that provide a favorable environment for cell growth and new bone tissue formation [181,197,198]. PCL fibers are a type of biomimetic nanofiber designed to mimic the properties of biological tissues and promote the regeneration of damaged tissues. Thanks to these characteristics, they have been FDA-approved for clinical applications. These fibers are produced through electrospinning, a technique that creates thin, porous structures, ideal for stimulating cell growth and promoting tissue regeneration. Studies have shown that this material supports osteochondral regeneration [199].

Clinical studies have explored various applications of PCL. Kim et al. used printed PCL scaffolds to repair caudal septal deviations in 20 patients undergoing septoplasty [200]. The results demonstrated good mechanical properties, biocompatibility, and ease of surgical handling, with improvements in nasal obstruction symptoms and septal morphology. At Xijing Hospital, Liu et al. [201] evaluated the use of 3D-printed PCL/TCP cages for lumbar interbody fusion in 22 patients. The results showed a bone fusion rate of 95.2% and significant clinical improvements [202].

However, PCL has significant limitations: it degrades very slowly inside the body [203,204,205] and its hydrophobicity can hinder cell promotion, adhesion, and infiltration [206]. As a result, currently there are few commercial PCL-based products for bone regeneration.

## 7. Composite Materials

Composite materials are obtained by combining materials with different physical and chemical properties. In these scaffolds, a continuous matrix hosts a discontinuous reinforcement. The continuous matrix is the main material that provides structure and support, while the discontinuous reinforcement consists of particles, fibers, or other dispersed elements within the matrix. This reinforcement helps improve both mechanical and biological properties. Compared to single-material scaffolds, composite scaffolds offer better biological performance and design flexibility. The choice of materials determining biocompatibility, biodegradability, and osteoconductivity [207]. Metals, bioceramics, and biopolymers are commonly employed due to their biocompatibility and biodegradability [4,5,107,208,209,210,211,212].

Although HA and β-TCP bioceramics are brittle, their applicability improves when combined with other biomaterials. Gao et al. [213] demonstrated that 0.5% silicon (Si) enhances osteoconduction and calcification. Fielding et al. [214] highlighted the improvement of mechanical properties with the addition of silicon oxide (SiO₂) and zinc oxide (ZnO) in β-TCP scaffolds.

Polymer-ceramic composites are promising alternatives for bone substitution and regeneration [215,216]. β-TCP and HAp, coated with biocompatible and biodegradable polymers such as PLGA, PGA, PCL, and PLA, form interconnected microstructures [217]. HA and PLA are widely used in scaffold production [218,219]. HA, the main inorganic component of bone, is highly biocompatible, osteoconductive, and promotes cell proliferation. PLA has an adjustable degradation rate [220,221], allowing adaptation to bone defect healing and providing support until complete tissue regeneration [207]. There is a substantial difference in chemical and physical properties between the polymeric and ceramic phases, which results in poor bonding strength. Coupling agents are used to improve the weak bond between bioceramics and biopolymers. These agents have two functional groups: one binds to the polymer, and the other attaches to the ceramic, strengthening the connection [222].

The integration of synthetic materials with xenografts is emerging as an innovative and promising approach, with applications in clinical settings. This combination allows for the exploitation of the complementary qualities of both materials: xenografts provide a natural biological matrix characterized by high biocompatibility and osteoconductive properties, while synthetic materials improve mechanical performance, regulate the resorption process, and reduce the risk of pathogen contamination [223]. Further developments in this field involve the introduction of resorbable polymers to enhance the properties of xenobiotic biomaterials. These polymers can be designed to locally release bioactive molecules, including growth factors, antimicrobial peptides, and osteoinductive drugs. In addition to promoting bone regeneration, this strategy helps prevent postoperative infections and modulate the local inflammatory response. Some studies suggest that these next-generation biomaterials could offer superior clinical outcomes compared to traditional xenografts, in terms of integration with surrounding tissues, structural strength, and regenerative capacity [223]. However, at present, a limited amount of these innovative products are available, included SmartBone^®^ ORTHO [22,224,225,226], Hypro-Oss^®^ ORTHO [227], and Tutobone^®^ [228]. Their characteristics and main clinical applications are described in Table 5.

**Table 5:** Summarizes commercially bone grafts and biomaterials, detailing their material type, description, and primary clinical applications. It includes xenografts, bioceramics, calcium sulfate-based materials, bioactive glasses, PLA, PGA, PLGA, and composite xenograft.

Commercial Name	Material Type	Description and Application
CANCELLO-PURE^®^	Xenograft (Bovine)	It is a xenogeneic bone graft derived from bovine, processed through decellularization to reduce the risk of immune rejection. It has been used in foot and ankle reconstruction procedures, such as Cotton osteotomy and Evans osteotomy [44]. However, some studies have reported high rates of graft non-union and failure to integrate, leading to its discontinuation in certain institutions.
Kiel-Surgibone^®^	Xenograft (Bovine)	It is a bovine bone graft treated with specific methods to remove antigenic components while preserving the bone’s mineral structure. It has been used in spinal fusion surgeries, such as anterior cervical discectomy and fusion (ACDF), showing fusion rates comparable to autografts in various clinical studies [44].
BIO-GEN^®^	Xenograft (Equine)	It is a heterologous bone substitute obtained from equine bone, processed through deproteinization and sterilization to remove antigenic components. It is used in oral and maxillofacial surgery for guided bone regeneration, supporting new bone formation thanks to its porous structure and biocompatibility [47].
Apaceram^®^	Bioceramic (HAp)	It is primarily used in orthopaedics and maxillofacial surgery for bone regeneration. It is employed to treat bone defects, facilitating the formation of new bone tissue and improving integration with the surrounding bone [96,97].
Straumann^®^ BoneCeramic™	Bioceramic (HAp)	It is applied in orthopaedics, especially for the replacement of bone portions damaged by trauma or degenerative diseases. Its use promotes bone growth and integration with natural bone tissue [98].
Cerament^®^ Bone Void Filler	Bioceramic (HAp)	It is used to fill bone defects and promote bone regeneration. It is employed to treat fractures and bone defects caused by malformations or infections, with mechanical resistance that increases over time as the new bone replaces the material [99,100].
Nanostim^®^	Bioceramic (Nanocrystalline HAp)	It is a form of nanocrystalline HAp. It is used in the medical field for bone regeneration, implant osseointegration, the treatment of osteoporosis, and, in some cases, in aesthetic medicine for soft tissue filler treatments [101].
KeraOs^®^	Bioceramic (β-TCP)	It is used in orthopaedics for bone regeneration, particularly in cases of bone defects and fractures. This material promotes osteointegration and bone recovery, supporting the consolidation of fractures and bone growth in orthopaedic procedures, such as the reconstruction of compromised bone areas or the treatment of complex fractures [101].
Vitoss^®^	Bioceramic (β-TCP)	It is used in orthopaedics to stimulate bone growth in complex fractures or significant bone defects, such as those caused by trauma or pathology. It is also employed to fill damaged or compromised areas, providing biologically active support during the regeneration process [108].
SynthoGraft™	Bioceramic (β-TCP)	Promotes bone regeneration and controlled resorption, making it useful for treating bone defects or fractures where a material capable of gradually integrating with surrounding bone tissue is needed. It is utilized in orthopaedic surgical procedures requiring safe and gradual regeneration of compromised bone tissue [109,110].
Maxresorb^®^	Bioceramic (BCP)	Features a porous structure that enhances cell adhesion and bone regeneration, making it suitable for addressing bone defects in orthopaedic contexts. It promotes bone repair in areas where regeneration is required and supports the healing process by providing a favorable scaffold for new tissue formation [111].
MBCP+ (Micro-Macroporous Biphasic Calcium Phosphate)	Bioceramic (BCP)	It is used in implantology to stabilize implants and promote osteointegration. It is utilized in orthopaedics for stabilizing implants and encouraging osteointegration in cases of bone defects. It responds to different clinical needs, including the filling of bone defects and other surgical applications that demand regenerative support [112].
Ceraform^®^	Bioceramic (BCP)	It is used in implantology, periodontology, and maxillofacial surgery to regenerate bone and fill defects. Its bioactive structure promotes osseointegration and new tissue formation, making it an effective material for procedures requiring defect repair or bone stabilization [113].
Bondbone^®^	Calcium Sulfate	In orthopaedics, it is used to treat bone defects, including peri-implant defects, and to support the healing of compromised bone structures. The controlled resorption of Bondbone^®^ allows for a gradual release of calcium, which stimulates new bone growth, this makes it especially useful for filling bone defects and preparing surgical sites while encouraging integration with native bone tissue [114].
EthOss^®^	Calcium Sulfate + β-TCP	It is particularly effective in preserving bone volume following trauma or surgical interventions, preventing bone loss, and maintaining the stability of the surrounding skeletal structure. In orthopaedic contexts, EthOss^®^ is frequently used to reconstruct complex bone defects, providing temporary mechanical support while stimulating bone growth. Its ability to maintain structural integrity during the healing process makes it ideal for preparing affected areas for subsequent interventions, such as implants or prosthetics [115].
NovaBone^®^	Bioactive Glass	FDA-approved and in Europe for orthopaedic use in 2000, is a particulate BG used in non-weight-bearing defects. It showed promising results compared to iliac crest autografts [122].
Medpor^®^-Plus	Bioactive Glass	Plus is the first BG composite for ocular bone grafts, developed to stimulate fibrovascular growth and vascularization, FDA-approved in 2002 [123].
BoneAlive^®^	Bioactive Glass	Uses S53P4, a silicate-based composition, with a slower dissolution rate compared to 45S5. It has been effective in bone infections and spinal fusions [124].
Glassbone^®^	Bioactive Glass	FDA-approved in 2008 for orthopaedic applications, showed 90% recovery in one year with a 60% reduction in pain [125].
Cortoss^®^	Bioactive Glass	FDA-approved in 2009, uses a polymer matrix to stimulate osteointegration. Clinical studies showed it provides greater pain relief than PMMA cements [126].
StronBone™	Bioactive Glass + Strontium	A strontium-doped bioactive glass, is approved in Europe but not the U.S. It inhibits osteoclast activity and stimulates osteogenesis [127].
Glace^TM^	Bioactive Glass	A fiber-glass composite for cranial and maxillofacial implants developed in 2014, weaves fibers into resin for enhanced mechanical properties [128].
OssiMend^®^ Bioactive	Bioactive Glass + Col 1 + apatite	It is a combination of 45S5 bioactive glass, type 1 Col, and carbonated apatite for bone defect repair, was FDA-approved in 2019. It promotes remineralization and dissolves faster than pure 45S5 glass [129]. It is still undergoing trials with 120 patients [130]
Signafuse^®^	Bioactive Glass + biphasic mineral	Is a composite of bifasic minerals and BG for bone grafting, was FDA-approved in 2020 and shows higher bone fusion rates than traditional materials [131].
LactoSorb^®^	Polymer (PLA)	It is widely used in osteosynthesis for maxillofacial surgery. This device consists of resorbable plates and screws, employed for the stabilization of mandibular and craniofacial fractures, as well as in orthognathic surgery. The degradation process of LactoSorb^®^ occurs within 12–18 months, eliminating the need for surgical removal once bone healing is complete [189].
Neoveil^®^	Polymer (PGA)	It is a 100% PGA membrane, used for GBR, in oral surgery and implantology. Its primary function is to protect and guide the growth of new bone tissue, making it particularly useful in post-tooth extraction procedures or sinus lift surgeries, where it promotes effective osseointegration [190].
Ethisorb^®^	Polymer (PLGA)	It is as a fixation device for maxillofacial and orthopaedic surgery. Composed of resorbable plates and screws, it is used to stabilize mandibular and zygomatic fractures, providing adequate mechanical support for approximately 6–12 months, after which the material is completely resorbed [191].
RapidSorb^®^	Polymer (PLGA)	Employed in cranio-maxillofacial fixation, with a particular application in post-surgical stabilization for pediatric procedures and reconstructive surgery. This device provides temporary support, essential for ensuring proper bone healing without requiring removal [192].
SmartBone^®^ ORTHO	Composite (Bovine Xenograft + Bioabsorbable Polymers + Col Fragments)	It is an innovative bone substitute developed for bone regeneration in orthopaedic, trauma, oncological, and spinal surgery. This hybrid structure promotes rapid growth of bone cells within the material, ensuring optimal integration and complete replacement with natural bone within 1–2 years. It is indicated for various orthopaedic applications, including: filling or reconstruction of acetabular bone during arthroplasty and revisions, after wedge osteotomies, filling of cavities post-curettage, reconstructions after bone tumor resections, and vertebral body replacement in spinal surgery [22,221,222,223].
Hypro-Oss^®^ ORTHO	Composite (Bovine Xenograft + HAp + Col)	It is a natural bovine bone material. Each granule consists of 30% Atelo-Col Type I and 70% HAp. Hypro-Oss^®^ is produced by using atelopeptidation and lyophilization processes. It is used for the permanent filling or reconstruction of antiseptic bone defects [224].
Tutobone^®^	Composite (Bovine Xenograft + Col)	It is a solvent-preserved cancellous bovine bone material. The production process involves the use of biochemical solvents that remove lipids, proteins and prions from the graft, but preserve Col and mineral compound. In this way the bone is sterilized and preserved for implantation, however, the biomechanical and structural integrity of the graft is maintained. The function of this graft is temporary structural support, integration in the surrounding bone, bioresorption and replacement with vital bone [225].

## 8. Specific Applications

### 8.1. Biomaterials for Reconstructive and Regenerative Surgery

Numerous clinical studies examine the use of bone grafts in foot and ankle surgery, where bone regeneration is crucial for treating conditions such as failed joint fusions, complex deformities, and non-healing fractures. These studies describe various available options, including autologous bone, allografts, and synthetic substitutes. The research reports that in joint fusions, the use of bone grafts has improved consolidation rates up to 90% [229].

The clinical use of bone grafts in spinal surgery is also well-documented, with a particular focus on vertebral fusion procedures. The authors emphasize that autologous bone, considered the gold standard, ensures effective fusion due to its osteogenic properties. However, its harvesting involves complications such as donor site pain and morbidity. As an alternative, synthetic substitutes, such as ceramic materials and bioactive matrices, provide excellent osteoconduction but lack intrinsic osteoinductive activity. In patients undergoing spinal fusion, the use of combinations of autologous bone and substitutes has led to a clinical success rate of 90%, with a significant reduction in healing time [230].

As a particular field of application, biomaterials play an important role in the tendon/ligament-to-bone healing process that poses a formidable clinical challenge due to the complex structure, composition, cell population and mechanics of the interface. With rapid advances in tissue engineering, a variety of strategies including advanced biomaterials, bioactive growth factors and multiple stem cell lineages have been developed to facilitate the healing of this tissue interface [92].

In recent years, bone reconstructive surgery in pediatric patients has made enormous progress thanks to the introduction of advanced biomaterials and innovative techniques such as 3D printing. This technology has significantly improved surgical precision, reduced operating times, and optimized post-operative outcomes. Additionally, 3D-printed anatomical models have enhanced surgical planning and communication with the families of pediatric patients [231].

Defects in long bones also represent an unmet clinical need in children. To date, autologous bone grafts harvested from different regions of the body, such as the fibula, represent the state of the art. One of the most significant developments concerns the use of vascularized fibular grafts, a particularly useful technique in limb salvage surgery for oncological conditions or severe trauma. By transferring a fibula segment along with its vascularization, surgeons have been able to reconstruct damaged bone segments, ensuring good graft integration and functional recovery. Despite some complications, such as infections and graft fractures, most children have achieved satisfactory long-term results [232].

Another widely studied approach is distraction osteogenesis, a technique used to lengthen bone segments or correct deformities with bioactive scaffolds or osteoconductive matrices. Data collected from pediatric patients treated with this method confirms that it allows for satisfactory bone growth and functional improvement. However, complications such as infections and joint stiffness must be carefully managed to optimize patient recovery [233].

Biological reconstruction, essential in managing bone defects following sarcoma resection, leverages the bone’s regenerative capacity through grafts and biological scaffolds. These approaches enhance osseointegration, reduce postoperative complications, and optimize functional outcomes. An interesting strategy for bone reconstruction after sarcoma resection involves biological scaffolds made from Col or HAp-based matrices, often enriched with growth factors like BMPs to stimulate bone tissue regeneration [234].

### 8.2. Biomaterials for Drugs Delivery

Drug delivery scaffolds represent a promising innovation in the treatment of bone diseases, with the potential to enhance therapeutic efficacy and bone regeneration.

A particular application of biomaterials is the treatment of osteomyelitis, a severe bone infection. These materials are typically composed of calcium sulfates, calcium phosphates, or bioactive glasses loaded with antibiotics. BG S53P4 combined with antibiotics has been used in the treatment of osteomyelitis. Its bioactive properties promote bone regeneration and integration with surrounding tissue, while its ability to release calcium and silicon ions aids healing. Additionally, the combination with antibiotics allows to treat bacterial infections and reduce the risk of recurrence [235]. The use of bone substitutes impregnated with antibiotics has shown superior results compared to conventional management in the treatment of osteomyelitis. This method has led to higher healing rates and a reduction in complications [236].

The treatment of osteoporosis is not limited to the use of medications to increase bone density and can also include surgical interventions, such as the use of bone grafts and bone substitutes. These materials are employed to repair or regenerate damaged bone tissue or to fill bone defects caused by fractures. In particular, CaP cements are valued for their biocompatibility and mechanical stability, making them ideal for filling bone defects in osteoporotic patients. The combined use of CaP with bisphosphonates has proven effective in reducing the risk of secondary fractures. Clinical studies show good bone integration of CaP, with applications particularly useful for the treatment of vertebral fractures [237]. The use of alendronate treatment on bone substitute materials, such as HAp and β-TCP, has shown significant improvements in the integration of bone substitutes into the surrounding tissue, leading to increased bone mineral density and more effective bone tissue regeneration. The combined approach of drugs and bone substitutes has shown good results even in conditions of severe bone demineralization [238]. Additionally, local treatment with parathyroid hormone PTH-34 and a whitlockite and β-TCP composite on bone regeneration in osteoporotic conditions has significantly stimulated osteoblastic activity, promoting faster bone regeneration. The composite improved bone microarchitecture and the mechanical strength of the newly formed bone [239].

Additionally, some multifunctional scaffolds are designed to release drugs or antibiotics, preventing tumor recurrences and infections, and promoting regeneration [234]. Drug delivery scaffolds represent an innovative strategy for osteosarcoma treatment, enabling targeted and controlled drug release directly at the tumor site, reducing systemic side effects, and ensuring precise dosage control. CS has been used to deliver doxorubicin, while gelatin has been combined with nanoparticles for the controlled release of chemotherapeutic agents. PLGA has been employed for the prolonged administration of methotrexate or doxorubicin. Composite scaffolds have proven particularly effective in both tumor cell destruction and bone regeneration, as seen in combinations such as CS with HAp or PLGA with carbon nanotubes [240].

### 8.3. Biomaterials for PRP and BMAC Delivery

Various biomaterials have been used to carry PRP (platelet-rich plasma) and BMAC (bone marrow aspirate concentrate) to enhance their effects and stimulate cell regeneration. The most commonly used materials in this field are organic scaffolds and both natural and synthetic polymers [241].

PRP contains growth factors in addition to platelets, such as platelet-derived growth factor (PDGF), transforming growth factorβ (TGF-β), basic fibroblast growth factor (bFGF), endothelial growth factor (EGF), and vascular endothelial growth factor (VEGF), which have an important effect on tissue repair and the proliferation and differentiation of mesenchymal stem cells (MSCs) [242].

A study on 60 patients showed that the use of autologous bone graft with PRP in maxillary sinus augmentation led to greater bone density and integration compared to the group without PRP. Another study on 45 patients confirmed that PRP accelerates bone regeneration and reduces graft volume loss compared to heterologous grafts without PRP, highlighting PRP’s key role in improving bone healing [243]. Another study compared PRP and platelet-rich fibrin (PRF) in bone regeneration in children, both combined with synthetic materials such as nanocrystalline HAp and β-TCP. PRF and PRP were both effective in promoting bone healing [244]. Finally, a study on spinal fusion demonstrated that the combination of PRP and artificial bone based on hydroxyapatite and collagen (HAp/Col) enhances bone formation and mechanical stability [245].

The use of BMAC (Bone Marrow Aspirate Concentrate) in the orthopaedic field is still recent, compared to PRP, but the results are very promising. Although progenitor cells have been used for a long time in clinics, BMAC is not only composed of progenitor cells, but also has a large quantity of growth factors [241]. BMAC is a concentrate of bone marrow aspirate, rich in mesenchymal stem cells, growth factors, and cytokines, which promote tissue regeneration and bone and cartilage healing. For this reason, it is used in orthopaedics, regenerative surgery, and dentistry to enhance tissue repair, especially in combination with bone grafts or surgical procedures [246]. A recent study suggests that adding BMAC to lateral meniscus allograft transplantation (MAT) may enhance integration and regeneration, accelerating healing and improving joint stability. The presence of mesenchymal stem cells could also help reduce meniscal extrusion [247]. A trial on 80 patients evaluated the effect of BMAC in anterior cruciate ligament (ACL) reconstruction using a bone–patellar tendon–bone (BTB) graft. At three months, Magnetic Resonance Imaging (MRI) showed higher metabolic activity in the BMAC group, suggesting a faster transformation of the graft into a functional ligament. At nine months, patients treated with BMAC exhibited excellent knee function, indicating that BMAC may promote a quicker recovery [248].

The application of a biomaterial is not only that of a therapeutic carrier of PRP and BMAC, but also as a functional regenerative scaffold for cell integration, proliferation, and differentiation that can expedite macroscale musculoskeletal tissue healing [241].

## 9. Discussion

Bone regeneration, with applications in orthopaedics, oncology, and reconstructive surgery, represents one of the most significant challenges in the medical field, particularly for large critical defects or in clinically compromised conditions. The growing need for effective bone reconstruction solutions has led to the development of numerous therapeutic strategies, including the use of autologous, allogenic, and xenogenic bone grafts, natural and synthetic biomaterials, and composite materials, each with specific advantages and limitations. The choice of the most appropriate treatment depends on various factors, such as the size of the bone defect, the patient’s clinical condition, and the availability of substitute materials.

Autologous grafts, considered the gold standard, offer excellent biocompatibility due to their ability to integrate and their osteogenic, osteoinductive, and osteoconductive properties. However, their application is limited by tissue availability, donor site morbidity, and postoperative complications, making complementary approaches necessary for large defects.

Allogenic grafts represent a useful alternative, especially in the form of demineralized bone matrix (DBM), which, while possessing osteoinductive capabilities, is often penalized by sterilization treatments that reduce its biological effectiveness. Immunogenic complications, the risk of disease transmission, and often insufficient supply further limit their widespread use.

Natural-derived materials, such as Col, HA, and CS, offer excellent biocompatibility and biodegradability. These biomaterials are used in membranes, hydrogels, and sponges, improving bone tissue regeneration. However, they have intrinsic limitations, such as low mechanical strength and rapid degradation, which can be mitigated through functionalization with nanoparticles or synthetic polymers. Alg and silk, for example, have shown promising applications in bone regeneration but require further studies to optimize their clinical use.

Synthetic biomaterials, including bioceramics, bioactive glasses, metals, and organic polymers, have expanded therapeutic options. Bioceramics, such as HAp and TCP, exhibit excellent osteoconductive properties, though they are limited by their fragility and poor moldability. Nanostructured variants have improved their stability and regenerative capacity. Bioactive glasses, known for their osteoinductive and antibacterial properties, are widely used in spinal fusion and cranio-maxillofacial regeneration but suffer from mechanical limitations and difficulties in controlling dissolution rates. Metal alloys, such as titanium, magnesium, and zinc, are a reference point for orthopaedic applications due to their strength and biocompatibility. Biodegradable metals, such as magnesium and zinc alloys, offer the advantage of eliminating the need for a second surgical removal, but their degradation rate and loss of mechanical strength require optimization. Synthetic organic polymers, such as PLA, PGA, PLGA, and PCL, allow for a high degree of customizing mechanical and biological properties. This makes them ideal for applications combined with bioactive factors or nanoparticles to enhance osseointegration.

Xenobiotic materials, derived from animal tissues, represent a versatile option. Among xenogenic grafts, those of bovine origin are among the most widely used in clinical practice. Bovine grafts are obtained through deproteinization and sterilization processes, which remove antigenic components while maintaining the mineral structure and porosity of natural bone. Their processing eliminates cellular components, reducing the risk of rejection, and preserves a highly osteoconductive bone structure. However, despite numerous advantages, some limitations persist, such as the risk of inflammatory reactions in more sensitive patients. Current research is exploring new technologies to improve the effectiveness of these materials, including their combination with growth factors and stem cells to enhance osteoinductive capacity.

In recent years, research has focused on innovative approaches to enhance the effectiveness of biomaterials used for bone regeneration, including the combined use of bioengineered scaffolds with bone morphogenetic proteins, platelet-derived growth factors, bone marrow aspirates, and mesenchymal stem cells. Additionally, incorporating antitumor agents, antibiotics, osteoinductive agents, and anti-osteoporotic drugs into scaffolds has improved bone healing in conditions such as sarcomas, osteoporosis, and osteomyelitis, while also reducing infectious and metabolic complications. Furthermore, technological innovations such as 3D printing and the development of multifunctional composite materials are opening new possibilities for personalized treatments, improving clinical outcomes.

Despite significant progress, considerable challenges remain. One of the main difficulties is scaffold vascularization, which is essential to ensure adequate cell survival and integration with host tissue. Additionally, controlling biomaterial degradation remains critical: materials that resorb too quickly can compromise implant structural stability, while those that persist too long can hinder bone remodeling. Another significant challenge is the standardization of materials, which is necessary to ensure uniform clinical outcomes and treatment safety. Currently, variability in biomaterial production can influence mechanical and biological properties, making it difficult to reproduce reliable results. Implementing advanced production protocols could improve the quality and consistency of bone grafts. An innovative approach lies in the development of more advanced 3D printing, enabling the creation of customized scaffolds with a microstructure that faithfully replicates the architecture of natural bone. The use of artificial intelligence and computational models could facilitate implant customization and clinical outcome prediction, allowing for a more targeted and effective approach.

Bone regeneration is a constantly evolving field, with promising developments in bioactive material design and innovative engineering strategies. The adoption of advanced biomaterials and combined therapies offers new perspectives for treating complex bone defects, improving patients’ quality of life, and reducing the need for repeated surgical interventions.

## 10. Conclusions

Bone regeneration is an area of research in continuous evolution, with significant implications for orthopaedics, oncology, and reconstructive surgery. Current therapeutic strategies, ranging from autologous, allogeneic, and xenogeneic bone grafts to natural, synthetic, and composite biomaterials, offer numerous options for managing bone defects. However, each of these solutions presents specific advantages and limitations, making it necessary to carefully evaluate each case. Emerging technologies, such as 3D printing and the use of engineered biomaterials, are revolutionizing the field, enabling personalized solutions and improving clinical outcomes. Despite significant progress, challenges remain, including scaffold vascularization, material degradation control, and standardization of manufacturing processes. The adoption of advanced protocols and the use of artificial intelligence could help overcome these limitations, improving the quality and effectiveness of bone implants.

## Figures and Tables

**Table 1:** Described uses, advantages, and disadvantages of naturally derived biomaterials.

Material	Main Uses	Advantages	Disadvantages
Collagen (Col)	Membranes, sponges, hydrogels for bone regeneration, bioactive substance delivery (BMP-2, FGF-2)	Biocompatible and biodegradable	Limited mechanical strength, unpredictable resorption rates, rapid degradation, low stiffness
Gelatine (Gel)	Used in bone regeneration combined with other materials (Hap, PCL)	Biocompatible, biodegradable, enhanced with other materials	Rapid degradation, poor mechanical strength
Alginate (Alg)	Hydrogels used for cells and bioactive molecule delivery (BMP, TGF-β), cartilage and bone tissue regeneration	Biocompatible, biodegradable, chemically modifiable, minimally invasive injection	Low mechanical strength, lack of cell-binding sites, structural disintegration, low in vivo degradation
Silk Fibroin (SF)	Scaffolds for skin, bone, cartilage, blood vessels	Biocompatible, biodegradable, low immunogenicity	Poor cell recruitment capabilities, fragile structures
Hyaluronic Acid (HA)	Used in bone regeneration combined with other materials (Gel, Col, BCP, SF, HAp)	Biocompatible, biodegradable, interacts with cells and bone-forming growth factors	Poor mechanical properties, low tensile and compressive strength
Chitosan (CS)	Nanofibers, used in bone regeneration, controlled delivery systems for growth factors and drugs	Biocompatible, biodegradable, antibacterial, high cell adhesion	High degradation rate, low chemical-physical stability
Polyhydroxyalkanoates (PHA)	Micro-channeled membranes, nanofibers and composite materials with carbon nanotubes used in bone regeneration	Bioactivity, biocompatible, pH stability during degradation ensures immune system tolerance	High batch-to-batch variation, difficulty in large-scale production

**Table 2:** Uses, advantages, and disadvantages of bioceramics.

Material	Main Uses	Advantages	Disadvantages
Hydroxyapatite (HAp)	Porous, pellet, and nanocrystalline forms used for bone tissue replacement	Strong bone bonding, osteoconductive, promotes stable fixation	Low degradability and fragility
Tricalcium Phosphate (TCP) (β-TCP, α-TCP)	Bone defect treatment caused by trauma and tumor, β-TCP used for filling cavities and for treating congenital or degenerative lesions	Highly osteoconductive, resorbable within weeks,	Low tensile strength, unsuitable for load-bearing areas, rapid degradation can outpace new bone formation
Biphasic Calcium Phosphate (BCP) (HAp + TCP)	Bone grafting in large defects and weight-bearing areas	Combines stability of HAp with resorption of TCP, tunable degradation rate, osteoconductive	Balancing resorption rates is complex, mechanical properties vary based on composition
Calcium Sulfate	Bone defect filler, combined with other CaP (β-TCP) to improve mechanical properties	Fastest resorption (4–12 weeks), low cost, easy preparation	Rapid degradation limits bone regeneration, weak mechanical properties, unsuitable for load-bearing applications

**Table 3:** Uses, advantages, and disadvantages of metallic materials.

Material	Main Uses	Advantages	Disadvantages
Stainless Steel	Joint implants, orthopaedic fixation	Strong, cost-effective	High elastic modulus, stress shielding, poor wear resistance, risk of metal ion release
Titanium (Ti)	Orthopaedic implants, 3D-printed implants used in spinal and hip surgery, bone tumor resection and complex post-traumatic skeletal defects treatment	Good biocompatibility, corrosion resistance, elasticity closer to bone	Biologically inert, requires surface modifications for better osteointegration, require a second surgery for removal after bone healing
Cobalt-Chromium (Co-Cr) Alloys	Orthopaedic implants, 3D-printed implants used in spinal and ankle surgery	High mechanical strength, excellent wear resistance	High rigidity, potential stress shielding, reduced bone integration
Magnesium (Mg) Alloys	Used for bone fracture fixation	Good biocompatibility, osteogenic potential, biodegradability, avoid secondary surgical procedures	Requires better mechanical stability and controlled degradation
Iron (Fe) Alloys	Used for bone fracture fixation	Gradual degradation in vivo, resorbable material, avoid secondary surgical procedures	Oxidation can lead to instability, mechanical strength loss during degradation
Zinc (Zn) Alloys	Used for bone fracture fixation	Stable mechanical support, gradual dissolution without residues, avoid secondary surgical procedures	Degradation rate may not match bone healing, mechanical strength loss during degradation
Bismuth (Bi) Alloys	Injectable bone fillers	Low melting point, in situ solidification for better clinical application	Limited studies on long-term safety and mechanical reliability
Tantalum (Ta)	Orthopaedic implants, 3D-printed implants used in knee and ankle surgery and complex post-traumatic skeletal defects treatment	Corrosion-resistant, elastic modulus similar to bone	High density, complex manufacturing process, slowly bone formation
Magnetostrictive Iron-Gallium (Fe-Ga) Alloys	Smart implants	Deformable under magnetic fields, potential for intelligent devices	Experimental stage, clinical applications still under study

**Table 4:** Uses, advantages, and disadvantages of polymer organic synthetic materials.

Materials	Main Uses	Advantages	Disadvantages
Polymethyl methacrylate (PMMA)	Spinal vertebroplasty, bone filler in primary tumors or metastatic patients, 3D printing of customized bioimplants for bone reconstruction	Easy to produce and handle Good biocompatibility Adequate mechanical strength and elasticity	Bioinert (not osteoinductive/osteoconductive) Heat sensitive during polymerization Risk of fragmentation and foreign body reaction
PMMA–CaP	Bone regeneration (studies as trabecular bone substitute on animal model)	Improved biocompatibility Better mechanical stability Promotes osteointegration	Clinical validation required
PMMA-BODBB	Bone reconstruction (studies on animal model)	Enhanced bioactivity and osteointegration Lower polymerization temperature Reduced toxicity from free radicals and toxic ions	Clinical validation required
PMMA-TaC	Advanced orthopaedic applications	Improved mechanical strength (>100 MPa) Improved radiopacity Biocompatibility maintained	Clinical validation needed
Polyglycolide (PGA)	Internal bone fixation, PGA membranes used in Guided bone regeneration (GBR), oral surgery, implantology	Biodegradable and biocompatible High thermal stability and sterilization resistance Supports bone regeneration	Low mechanical strength Rapid degradation with acidic byproducts (inflammation risk)
Polylactic Acid (PLA)	Guided bone regeneration (GBR) membranes, orthopaedic applications for low mechanically loaded implants, maxillofacial surgery	Biodegradable and biocompatible, good mechanical properties, molecular weight and crystallinity modifiable	Hydrophobic Low impact toughness Slow degradation with acidic byproducts (possible inflammation)
Polylactic-co-glycolic Acid (PLGA)	Drug delivery, bone fixation in cranio- maxillofacial and orthopaedic surgery, surgical stabilization for pediatric procedures and reconstructive surgery	Controlled degradation rate (by adjusting PLA/PGA ratio) Biocompatible, good cell adhesion and proliferation	Low mechanical strength Hydrophobicity Limited bioactivity Potential release of acidic byproducts
Polycaprolactone (PCL)	Bone and periodontal tissue engineering, nanofiber used in osteochondral regeneration, repair of caudal septal deviations, lumbar interbody fusion	High mechanical strength, scaffolds favorable for cell growth and bone formation, nanofibers stimulate cell growth and tissue regeneration	Slow degradation rate, hydrophobicity hinders cell adhesion, infiltration, and promotion

## Data Availability

The original contributions presented in this study are included in this article. Further inquiries can be directed to the corresponding authors.
